# The association between ultra-processed food consumption and health-related quality of life differs across lifestyle and socioeconomic strata

**DOI:** 10.1186/s12889-024-19351-7

**Published:** 2024-07-22

**Authors:** Somayeh Hosseinpour-Niazi, Mahdieh Niknam, Parisa Amiri, Parvin Mirmiran, Elaheh Ainy, Neda Izadi, Zahra Gaeini, Fereidoun Azizi

**Affiliations:** 1 Nutrition and Endocrine Research Center, Research Institute for Endocrine Sciences, Shahid Beheshti University of Medical Sciences, P.O. Box: 19395-4763, No. 24, A’rabi St., Yeman Av., Velenjak, Tehran, Iran; 2 Research Center for Social Determinants of Health, Research Institute for Endocrine Sciences, Shahid Beheshti University of Medical Sciences, P.O. Box: 19395-4763, No. 24, A’rabi St., Yeman Av., Velenjak, Tehran, Iran; 3 Department of Clinical Nutrition and Dietetics, Faculty of Nutrition Sciences and Food Technology, National Nutrition and Food Technology Research Institute, Shahid Beheshti University of Medical Sciences, Tehran, Iran; 4 Safety Promotion and Injury Prevention Research Center, Shahid Beheshti University of Medical Sciences, Tehran, Iran; 5 Endocrine Research Center, Research Institute for Endocrine Sciences, Shahid Beheshti University of Medical Sciences, Tehran, Iran

**Keywords:** Ultra-processed foods, Health-related quality of life, Lifestyle factors, Socioeconomic status

## Abstract

**Background:**

In this prospective study, we aimed to examine the association between ultra-processed foods and health-related quality of life (HRQoL) and to evaluate the effect of lifestyle and socioeconomic factors on this association.

**Methods:**

This study included 1766 adults (aged 18 to 78, 54.3% women), who took part in the Tehran Lipid and Glucose study. The Short-Form 12-Item Health Survey version 2 was used to determine HRQoL, which includes the physical component summary (PCS) and mental component summary (MCS) scores. Ultra-processed food consumption was assessed using a validated semi-quantitative food frequency questionnaire. Lifestyle (physical activity and smoking status) and socioeconomic factors (education level and employment status) were also determined. General linear models (GLM) were applied to estimate the mean (95% confidence interval) for MCS and PCS scores across the ultra-processed foods tertiles. Additionally, the effect of lifestyle and socioeconomic factors on the relationship between ultra-processed foods and HRQoL was examined using GLM.

**Results:**

The median consumption of ultra-processed foods was 11.9% (IQR: 8.2 to 16.8) of total energy intake. There was a significant inverse association between ultra-processed foods consumption and PCS, but not MCS, after adjustment for confounding factors. Significant interactions were observed between ultra-processed food consumption, sex, and occupation on PCS score (all *P* values < 0.001). The interaction test tended to be significant for smoking status, education levels, and physical activity levels. As ultra-processed food consumption increased, the PCS score significantly decreased in women (*P* = 0.043), low physical active subjects (*P* = 0.014), smokers (*P* = 0.015), and lower-educated individuals (*P* = 0.022). Non-employed individuals with higher ultra-processed food intake showed a decline in their PCS and MCS scores. While there was no significant difference in MCS score among different strata of lifestyle and socioeconomic status across tertiles of ultra-processed foods.

**Conclusions:**

Higher intake of ultra-processed foods was associated with poorer physical health, particularly among women, those with unhealthy lifestyles, and low socioeconomic conditions.

## Introduction

Ultra-processed foods products are heavily processed products, with little or no whole foodstuff. Given their high energy content, high sugar, high salt, and unhealthy fats, as well as their deficiency in fiber, proteins, and micronutrients, the majority of these foods are regarded as being of poorer nutritional quality [1, 2]. Globally, the consumption of ultra-processed foods products is rapidly rising and leads to obesity [3] and cardiovascular diseases [4]. Ultra-processed food consumption is also associated with other health aspects such as psychological and health-related quality of life (HRQoL), however, this aspect has been less studied. Consumption of these food products is associated with poor quality of life [5–9]. Most of these studies focus on adolescents, which limits their generalizability to adult populations. However, prospective studies have shown that decreased adherence to healthy dietary patterns such as the Dutch Healthy Diet index, high fiber diet, and vegetarian diet was associated with worse physical and mental health [10, 11], a finding that was not observed in others [12–14].

Moreover, ultra-processed foods consumption and HRQoL are likely influenced by various factors such as lifestyle and socioeconomic conditions. Socioeconomic differences in ultra-processed foods consumption [15–17] and HRQoL [18–20] have been reported in various countries. Additionally, unhealthy behaviors such as smoking and a sedentary lifestyle result in a significant decline in HRQoL over time [12, 21–23]. The observed association may be attributed to poor dietary choices, including increased consumption of alcoholic beverages, coffee, sweets, and ultra-processed foods [24–26]. Thus, socioeconomic status and unhealthy habits may affect both ultra-processed food consumption and HRQoL [10] and modify the association between quality of life and ultra-processed food consumption. However, few studies investigated these modifying effects. To our knowledge, only one study has examined the combined effects of smoking and ultra-processed foods on chronic diseases [27].

Therefore, undertaking the Tehran Lipid and Glucose Study (TLGS), a population-based cohort study, our objectives were to (1) assess the association between ultra-processed foods consumption, HRQoL, socioeconomic and behavior habits (smoking status and physical activity levels) and (2) determine if behavior habits and socioeconomic factors might modify the association between ultra-processed foods and HRQoL.

## Materials and methods

### Study design and participants

This prospective study is designed within the TLGS framework; a longitudinal population-based cohort study began in 1999 to investigate the risk factors for non-communicable diseases and promote a healthy lifestyle in Tehran’s urban population. Follow-up visits accrue at approximately 3-year intervals. The study’s examination cycles after the baseline examination (1999–2002) were: Phase 2 (2002–2005), Phase 3 (2005–2008), Phase 4 (2009–2011), Phase 5 (2012–2015), Phase 6 (2015–2018) and Phase 7 (2019–2021). Details of the study design and methodology have previously been published elsewhere [28].

In the current study, Phase 3 was considered as the baseline, which included 12,519 participants aged ≥ 3 years. Due to the cost, time-consuming, and complexity of gathering dietary data in large populations, a representative sample of 4920 participants was randomly selected to gather dietary information. Out of these, 3568 subjects completed the Food Frequency Questionnaire (FFQ). 196 subjects were excluded due to energy intake ≤ 800 kcal or ≥ 4200 kcal (*n* = 196) [29]. Subsequently, the remaining subjects (*n* = 3372) were followed up to Phase 7. Subjects aged ≤ 18 years (*n* = 194) and those lacking data regarding HRQoL (*n* = 1412) were excluded. Ultimately, this analysis included 1766 subjects.

This study was conducted according to the guidelines laid down in the Declaration of Helsinki, and all procedures involving human subjects/patients were approved by the Institutional Review Board of the Research Institute for Endocrine Sciences (RIES), Shahid Beheshti University of Medical Sciences, Tehran, Iran (IR.SBMU.MSP.REC.1401.462). Written informed consent was obtained from all subjects/patients.

### Dietary assessment

During face-to-face interviews with expert dietitians, the valid and reliable semi-quantitative FFQ was used to estimate dietary intake. The frequency of consuming each food item was recorded based on portion size over the past year. After that, the portion sizes were converted to grams. The Iranian Food Composition Table (FCT) was utilized to ascertain the amounts of macronutrient and micronutrient consumption [30].

In the current study, UPF is defined based on the NOVA food group classification system, which categorizes foods according to the extent and purpose of food processing [2]. UPF included hydrogenated fat, mayonnaise, margarine, potato chips, Puffs, hamburger, sausage, pizza, sugar-sweetened beverages, biscuits, cakes, candies, chocolates, ice cream, cocoa milk, crackers, Iranian confectionery (gaz, Sohan, halvah), and pastries (non-crème and creamy).

Of the 1766 participants at baseline, 683 completed all four FFQs, 222 completed three, 578 completed two, and 283 declined to complete the FFQ during the follow-up period. Missing values were imputed using the last observation carried forward method [31]. Regarding the crucial effect of recent dietary intakes on chronic disease events, dietary variable consumption was estimated using an alternative approach proposed by Hu et aln [31]. This approach is considered more important than the baseline measures, adds more weight to the recent diet, decreases within-subject variability, and assesses long-term diet.

Intake of dietary variables, collected using the FFQ, reported a valid estimate against multiple 24 recalls and between two FFQs [32, 33]. Moreover, the reliability, validity, and stability of the dietary patterns were reasonable based on the data collected from the FFQ over 8 years [34].

### Clinical and laboratory measurements

As the TLGS design, a standard questionnaire, including demographic information, age, education level, employee status, physical activity, smoking status, marital status, and a family history of type 2 diabetes was used and completed by a skilled interviewer. Subjects with a university degree were categorized into higher-educated individuals, while those with a degree lower than a diploma were classified into lower-educated individuals. Additionally, participants were divided into two groups based on economic status: (1) employed with income and (2) unemployed without income.

Weight was measured using a digital scale (Seca 707: range 0/0 –150/0 kg) with a sensitivity of 0.1 kg. Participants wore light clothing and no shoes. Height was measured using a tape meter while standing without shoes. BMI (kg/m^2^) was calculated by dividing weight by the square of height. Physical activity was evaluated using the Modifiable Activity Questionnaire (MAQ), which recorded the frequency and duration of physical activity over the past year [35]. Physical activity levels were reported as metabolic-equivalent (MET) minutes per week (MET-min/week) [36]. This value was used to categorize participants based on activities as low (< 3 MET hour-week) or medium/high (≥ 3 MET hour-week) PAL [37]. The reliability and convergent validity of the Persian version of the MAQ have been previously reported [38].

### Health-related quality of life

The HRQoL was evaluated using a reliable and validated Persian version of the short-form 12-item health survey version 2 (SF-12v2) [39]. This survey consists of two dimensions: physical and mental, each comprising four subscales. The physical subscales encompass physical functioning, role physical, bodily pain, and general health, while the mental subscales include vitality, social functioning, role emotional, and mental health. Each subscale score in the SF-12v2 ranges from 0 (indicating the poorest health condition) to 100 (indicating the best health condition). Additionally, the survey generates two summary scores: the physical component summary (PCS) and the mental component summary (MCS), which provide weighted summaries of each respective domain.

### Statistical analysis

Ultra-processed foods consumption was adjusted for energy by the residual method [40] and was modeled as tertiles. The normality of the distribution of variables was assessed by the Histogram and the Kolmogorov-Smirnov test. Characteristics of participants were expressed as mean ± standard error (SE) for continuous variables and percentages for categorical variables. General linear models were used to estimate the mean (95% confidence interval) for MCS and PCS scores and their components across the tertiles of ultra-processed foods. Two models were fit. The first model was adjusted for age, sex, BMI, and energy intake. The second model additionally adjusted for smoking status, physical activity levels, marital status, and education level.

Ultra-processed foods consumption had significant interactions with sex, and occupation on PCS scores in the multivariable model (all *P* values < 0.001); although the interaction test tended to be significant for smoking status, education levels, and physical activity levels (*P* interaction for smoking status = 0.071; *P* interaction for education levels = 0.063; *P* interaction for physical activity levels = 0.059). Therefore, we evaluated the effect of lifestyle factors and socioeconomic status on the association between ultra-processed foods and PCS scores. All statistical analyses were performed in SPSS version 15.0 (SPSS Inc., Chicago, IL, USA), and *P*-values less than 0.05 were considered statistically significant.

## Results

Of the 1766 individuals, the majority were women (54.3%, *n* = 959), had low physical active levels (44.6%, *n* = 788), were smokers (86.3%, *n* = 1524), had lower levels of education (1461, *n* = 82.7), and were not employed (55.5%, *n* = 980). The mean (SD) age and BMI were 39.4 years (13.3) and 26.7 (4.6), respectively. The median ultra-processed foods were 11.9% (IQR: 8.2 to 16.8) of total energy intake. The most common UPFs were hydrogenated vegetable oil (24.9%), biscuits (9.8%), cakes (9.3%), ice creams (9.0%), potato chips (8.3%), and mayonnaise (3.6%). Table 1 shows the baseline characteristics and dietary intakes of subjects according to the tertiles of ultra-processed foods. Subjects who consumed higher ultra-processed foods were mostly women, younger, smokers, unmarried, and had lower BMI. They also consumed more energy, fat, saturated fatty acids, mono-unsaturated fatty acids, polyunsaturated fatty acids, cholesterol, meat and processed meat, poultry and fish, and refined grains. Conversely, the consumption of carbohydrates, proteins, fiber, vegetables, fruits, whole grains, and dairy products decreased across the ultra-processed foods tertiles.

**Table 1:** Baseline characteristics of participants across tertiles of ultra-processed food

	Ultra-processed food			
	T1	T2	T3	*P* value
Range of intake (% of total energy)	≤ 9.0	9.1–15.3	≥ 15.4	
Median intake (% of total energy)	9.6	12.1	17.8	
Age (y)	43.6 ± 0.5	39.4 ± 0.5	35.3 ± 0.5	< 0.001
Female	367 (62.3)	310 (52.6)	282 (48.0)	< 0.001
Physical activity (MET hr.-week)	13.6 ± 0.8	13.0 ± 0.8	14.2 ± 0.8	0.593
Smoker	27 (4.6)	55 (9.7)	59 (10.5)	0.001
lower-educated individuals	92 (15.6)	96 (16.3)	117 (19.9)	0.113
Marital status, Married	484 (82.2)	439 (74.5)	399 (67.9)	< 0.001
Family history of diabetes, yes	103 (22.1)	84 (18.0)	88 (20.6)	0.548
BMI (kg/m^2^)	27.5 ± 0.2	26.5 ± 0.2	26.1 ± 0.2	< 0.001
*Dietary variables*				
Total energy (kcal/d)	2260 ± 27	2307 ± 27	2437 ± 27	< 0.001
Carbohydrate (% of total energy)	59.4 ± 0.2	58.2 ± 0.2	57.7 ± 0.2	< 0.001
Protein (% of total energy)	14.8 ± 0.1	14.2 ± 0.1	13.8 ± 0.1	< 0.001
Fat (% of total energy)	28.8 ± 0.2	30.4 ± 0.2	31.1 ± 0.2	< 0.001
SFA (% of total energy)	9.5 ± 0.1	9.9 ± 0.1	10.2 ± 0.1	< 0.001
MUFA (% of total energy)	9.7 ± 0.1	10.3 ± 0.1	10.5 ± 0.1	< 0.001
PUFA (% of total energy)	5.8 ± 0.1	6.2 ± 0.1	6.3 ± 0.1	< 0.001
Total fiber (g/d)	35.1 ± 0.7	31.9 ± 0.7	31.0 ± 0.7	< 0.001
Cholesterol (g/d)	200 ± 5	222 ± 5	247 ± 5	< 0.001
Vegetables (g/d)	319 ± 7	290 ± 7	266 ± 7	< 0.001
Fruit (g/d)	413 ± 10	357 ± 10	324 ± 10	< 0.001
Meat, and processed meat (g/d)	25.6 ± 1.3	28.6 ± 1.3	33.3 ± 1.3	< 0.001
Poultry and fish (g/d)	38.3 ± 1.2	38.6 ± 1.2	43.1 ± 1.2	0.012
Whole grain (g/d)	148 ± 4	125 ± 4	111 ± 4	< 0.001
Refined grain (g/day)	308 ± 6	314 ± 6	312 ± 6	0.742
Nuts (g/d)	7.5 ± 0.5	8.3 ± 0.5	8.9 ± 0.5	0.111
Legumes (g/d)	29.5 ± 1.2	30.2 ± 1.2	31.9 ± 1.2	0.315
Dairy products (g/d)	392 ± 8	363 ± 8	362 ± 8	0.020

MET, metabolic equivalent; BMI, body mass index; SFA, saturated fatty acids; MUFA, monounsaturated fatty acids; PUFA, polyunsaturated fatty acids. Values are mean ± SEM and number (%);Dietary variables were adjusted for energy intakes

The association between ultra-processed foods consumption and HRQoL scores is shown in Table 2. In terms of the physical component, physical functioning, role physical, and PCS all significantly decreased from 87.2, 81.2, and 48.6 in the first tertile of ultra-processed foods to 82.5, 78.2, and 47.7 in the tired tertile in model 1, respectively. These associations remained significant after further adjustment for confounding factors in model 2. Additionally, for the mental components, there were no significant associations observed for MCS and its subscale, except for social functioning, across ultra-processed foods consumption tertiles in both models 1 and 2.

**Table 2:** Multivariate adjusted mean (95% CI) for mental and physical components score and its component across tertiles of ultra-processed food products

	Model 1				Model 2			
	T1	T2	T3	*P*	T1	T2	T3	*P*
Physical functioning	**87.2 (85.3 to 89.1)**	**81.0 (79.1 to 82.9)**	**82.5 (80.6 to 84.4)**	**< 0.001**	**87.1 (85.1 to 89.0)**	**81.0 (79.1 to 82.9)**	**82.1 (80.1 to 84.0)**	**< 0.001**
Role physical	**81.2 (79.3 to 83.0)**	**77.2 (75.4 to 79.0)**	**78.2 (76.4 to 80.1)**	**0.008**	**81.1 (79.3 to 82.9)**	**77.1 (75.2 to 78.9)**	**78.1 (76.2 to 80.0)**	**0.008**
Bodily pain	79.4 (77.4 to 81.3)	77.3 (75.4 to 79.2)	77.7 (75.7 to 79.6)	0.300	79.1 (77.2 to 81.1)	77.4 (75.5 to 79.4)	77.6 (75.7 to 79.6)	0.427
General Health	48.1 (46.3 to 49.8)	46.6 (44.9 to 48.3)	46.9 (45.1 to 48.7)	0.484	47.7 (45.9 to 49.4)	46.6 (44.8 to 48.3)	46.5 (44.7 to 48.2)	0.574
**PCS**	**48.6 (48.0 to 49.3)**	**47.3 (46.6 to 47.9)**	**47.7 (47.1 to 48.4)**	**0.013**	**48.5 (47.9 to 49.2)**	**47.3 (46.6 to 47.9)**	**47.6 (47.0 to 48.3)**	**0.023**
Vitality	66.0 (63.9 to 68.1)	62.9 (60.9 to 64.9)	63.8 (61.7 to 65.8)	0.095	65.9 (63.8 to 68.0)	62.8 (60.8 to 64.9)	63.4 (61.3 to 65.5)	0.094
Social functioning	**83.9 (81.8 to 86.0)**	**79.4 (77.4 to 81.5)**	**82.5 (80.4 to 84.5)**	**0.008**	**84.0 (81.9 to 86.1)**	**79.6 (77.5 to 81.7)**	**82.0 (79.9 to 84.1)**	**0.014**
Mental health	72.2 (70.4 to 74.0)	69.5 (67.7 to 71.2)	69.7 (67.9 to 71.5)	0.063	72.2 (70.4 to 73.9)	69.5 (67.7 to 71.3)	69.4 (67.6 to 71.2)	0.057
Role emotional	78.2 (76.4 to 80.1)	75.8 (73.9 to 77.6)	75.4 (73.5 to 77.2)	0.072	78.2 (76.3 to 80.0)	75.9 (74.1 to 77.7)	75.2 (73.3 to 77.1)	0.071
**MCS**	49.6 (48.8 to 50.5)	48.5 (47.6 to 49.3)	48.7 (47.8 to 49.5)	0.143	49.6 (48.8 to 50.5)	48.5 (47.6 to 49.4)	48.5 (47.6 to 49.4)	0.130

Model 1 was adjusted for age, sex, BMI, and energy intake. Model 2 was further adjusted for smoking status, physical activity levels, marital status, education level. PCS, physical component summary; MCS, mental component summary

Multivariate adjusted mean (95% CI) for mental and physical components score and ultra-processed foods consumption by sex and different lifestyle strata is shown in Table 3. Men had higher scores for both physical and mental QoL than women. Smokers had higher PCS scores compared with non-smokers. Medium/high active individuals had higher PCS scores and its subscales including role physical, and general health, compared to low physical active subjects. There was no significant difference in MCS and its subscales scores between various groups of smoking status and levels of physical activity. Additionally, ultra-processed foods consumption was higher among men, smokers, and low-active subjects.

**Table 3:** Multivariate adjusted mean (95% CI) for mental and physical components scores according to sex and different lifestyle strata

	Men	Women	*P* value	Smokers	Non-smokers	*P* value	Low physical activity	Medium/high physical activity	*P* value
Physical functioning	88.5 (86.6 to 90.4)	79.3 (77.6 to 81.0)	< 0.001	83.8 (79.2 to 88.5)	84.2 (82.6 to 85.7)	0.903	81.6 (78.5 to 84.6)	84.9 (83.2 to 86.5)	0.061
Role physical	84.7 (83.0 to 86.5)	73.8 (72.2 to 75.4)	< 0.001	79.4 (75.0 to 83.8)	79.3 (77.9 to 80.8)	0.971	76.3 (73.5 to 79.2)	80.2 (78.7 to 81.7)	0.020
Bodily pain	83.5 (81.6 to 85.4)	73.5 (71.8 to 75.2)	< 0.001	77.6 (73.1 to 82.2)	79.6 (78.1 to 81.1)	0.435	77.4 (74.4 to 80.4)	80.0 (78.4 to 81.6)	0.134
General Health	50.1 (48.4 to 51.8)	44.7 (43.1 to 46.2)	< 0.001	45.8 (41.5 to 50.0)	49.0 (47.6 to 50.3)	0.166	46.2 (43.5 to 49.0)	49.4 (47.9 to 50.8)	0.051
PCS	49.6 (49.0 to 50.2)	46.4 (45.8 to 47.0)	< 0.001	48.1 (46.6 to 49.7)	47.2 (47.7 to 48.7)	0.049	47.1 (46.1 to 48.1)	48.5 (48.0 to 49.0)	0.019
Vitality	67.3 (65.3 to 69.3)	61.5 (59.7 to 63.4)	< 0.001	62.3 (57.4 to 67.2)	65.9 (64.4 to 67.5)	0.167	64.5 (61.3 to 67.7)	65.9 (64.2 to 67.6)	0.461
Social functioning	85.4 (83.3 to 87.4)	79.0 (77.1 to 80.8)	< 0.001	79.7 (74.7 to 84.7)	83.0 (81.3 to 84.6)	0.231	81.8 (78.6 to 85.1)	82.9 (81.1 to 84.6)	0.587
Mental health	74.8 (73.0 to 76.5)	66.8 (65.2 to 68.4)	< 0.001	67.7 (63.4 to 71.9)	72.0 (70.6 to 73.4)	0.062	70.2 (67.4 to 72.9)	72.0 (70.5 to 73.4)	0.260
Role emotional	80.4 (78.6 to 82.2)	73.1 (71.4 to 74.7)	< 0.001	75.5 (71.1 to 80.0)	77.2 (75.8 to 78.7)	0.479	76.1 (73.2 to 79.0)	77.3 (75.8 to 78.9)	0.461
MCS	50.5 (49.6 to 51.3)	47.6 (46.8 to 48.3)	< 0.001	47.6 (45.6 to 49.7)	49.6 (48.9 to 50.2)	0.087	49.1 (47.8 to 50.5)	49.5 (48.7 to 50.2)	0.658
Ultra-processed foods	10.4 (10.0 to 10.8)	10.0 (9.6 to 10.4)	0.197	11.4 (10.7 to 12.2)	10.0 (9.7 to 10.3)	< 0.001	10.4 (9.7 to 11.2)	9.1 (9.7 to 10.5)	0.040

Data was adjusted for age, sex, BMI, energy intake, smoking status, physical activity levels, marital status, education level. PCS, physical component summary; MCS, mental component summary

Table 4 shows the mean (95% CI) for mental and physical components score as well as ultra-processed foods consumption across different socioeconomic groups. Subjects with higher education exhibited significantly higher PCS scores compared to those with lower education, with no significant difference in terms of mental QoL. Employed subjects showed significantly higher scores for both physical and mental QoL compared to non-employed individuals. Ultra-processed food consumption was higher among those with lower education and non-employed participants.

**Table 4:** Multivariate adjusted mean (95% CI) for mental and physical components scores according to different socioeconomic strata

	Lower-educated individuals	Higher-educated individuals	*P* value	Non-employed	Employed	*P* value
Physical functioning	83.8 (82.2 to 85.4)	85.6 (82.2 to 89.0)	0.352	83.5 (81.4 to 85.6)	84.9 (82.5 to 87.4)	0.428
Role physical	79.0 (77.5 to 80.4)	81.1 (77.9 to 84.3)	0.234	78.5 (76.5 to 80.5)	80.4 (78.1 to 82.8)	0.265
Bodily pain	78.9 (77.3 to 80.4)	81.7 (78.4 to 85.1)	0.127	78.6 (76.5 to 80.7)	80.4 (78.0 to 82.9)	0.305
General Health	47.6 (46.1 to 49.0)	53.6 (50.5 to 56.6)	0.001	47.9 (45.9 to 49.8)	49.7 (47.4 to 51.9)	0.293
PCS	47.9 (47.4 to 48.5)	49.2 (48.1 to 50.4)	0.040	47.3 (47.0 to 48.6)	48.5 (47.7 to 49.3)	0.041
Vitality	65.2 (63.5 to 66.9)	67.2 (63.7 to 70.8)	0.311	63.1 (60.9 to 65.4)	68.7 (66.1 to 71.3)	0.004
Social functioning	82.7 (81.0 to 84.3)	82.5 (78.9 to 86.1)	0.950	81.2 (79.0 to 83.5)	84.4 (81.8 to 87.1)	0.105
Mental health	71.4 (70.0 to 72.9)	72.2 (69.1 to 75.3)	0.656	71.0 (69.1 to 72.9)	72.3 (70.0 to 74.5)	0.454
Role emotional	76.6 (75.1 to 78.1)	78.9 (75.7 to 82.1)	0.216	75.9 (73.8 to 77.9)	78.6 (76.2 to 80.9)	0.117
MCS	49.3 (48.6 to 50.0)	49.7 (48.2 to 51.2)	0.633	48.8 (47.8 to 49.7)	50.2 (49.1 to 51.3)	0.084
Ultra-processed foods	10.9 (10.3 to 11.6)	10.0 (9.7 to 10.3)	0.011	10.6 (10.2 to 11.0)	9.9 (9.5 to 10.2)	0.012

**Fig. 1:**
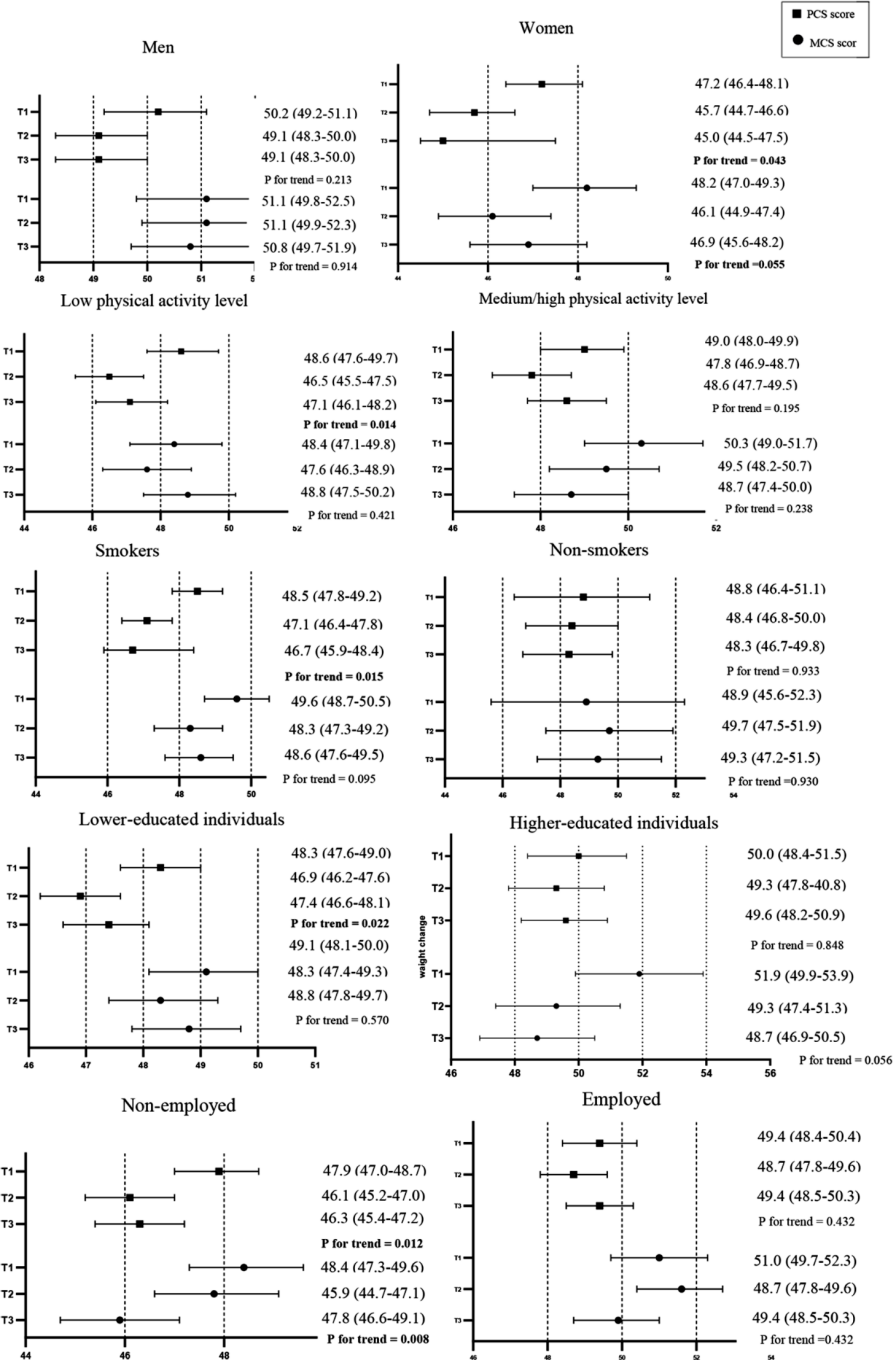
Multivariable mean (95% confidence interval) of the association between ultra-processed foods, physical component summary (PCS), and mental component summary (MCS) scores, stratified by sex, lifestyle, and socioeconomic status. Data were adjusted for age, sex, BMI, and energy intake, smoking status, physical activity levels, marital status, education level

Figure 1 shows the adjusted mean (95% CI) for PCS and MCS in relation to ultra-processed food consumption across different categories of sex, lifestyle factors, and socioeconomic status. Significant interactions were observed between ultra-processed foods consumption and sex, occupation on PCS score (all *P* value < 0.001); the interaction test tended to be significant for smoking status, education levels, and physical activity levels (*P* interaction for smoking status = 0.071; *P* interaction for education levels = 0.063; *P* interaction for physical activity levels = 0.059). As ultra-processed foods consumption increased, the PCS score significantly decreased in women, but not in men. There were no significant differences in MCS scores across ultra-processed food intake tertiles for both men and women. Among physically inactive subjects, higher ultra-processed food intake decreased the PCS score, while this association was not observed among physically active subjects. Ultra-processed foods consumption did not show an association with MCS score after adjustments for confounding factors, in both active and non-active subjects. Furthermore, a higher intake of ultra-processed foods was associated with a lower PCS score among smokers, but not among non-smokers. This association was not observed for MCS in different states of smoking status. Subjects with a lower-educated group showed a significant decrease in PCS score with increased ultra-processed foods consumption. There was no difference in the MCS score among the lower-educated group and in MCS and PCS scores among the higher-educated group across ultra-processed foods tertiles. Non-employed individuals with higher ultra-processed foods intake showed a decline in their PCS and MCS scores, while there was no significant difference in MCS and PCS scores among employed individuals across tertiles of ultra-processed foods.

## Discussion

The current study showed a higher ultra-processed food consumption was associated with worse physical, but not mental HRQoL after controlling for potential confounding factors. Furthermore, a high socio-economic status, encompassing high education and employment, was linked to enhancing physical, but not mental HRQoL. In terms of lifestyle, it was observed that being a smoker and being physically active were associated with improved physical health. Moreover, we found that the association between ultra-processed foods and physical health was modified by lifestyle and socioeconomic factors. The inverse associations were more pronounced among women, individuals with low socioeconomic status, smokers, and those with low physical activity levels. However, ultra-processed food consumption did not show an association with MCS score after adjustments for confounding factors in different strata of socioeconomic and lifestyle factors, except among employment subjects.

In the current study, as ultra-processed food consumption increased, PCS decreased, but MCS remained unchanged. A tendency for lower quality of life was found with increased ultra-processed food consumption among adolescents [8] and those with coeliac diseases [9]. Furthermore, a nutritional intervention that led to less consumption of ultra-processed foods consumption resulted in improved HRQoL among overweight/obese adolescents [5–7]. However, in later studies, a significant reduction in emotional scores following nutritional intervention was observed [5, 7]. Most of these studies are focused on adolescents, making them less generalizable to adult populations. To the best of our knowledge, no study has investigated the relationship between ultra-processed food consumption and quality of life in a population-based prospective study. However, our findings align with those of the Iowa Women’s Health Study, the Nurses’ Health Study [14], and AusDiab [12, 41], which found no statistically significant associations between MCS score and dietary fiber [41, 42], plant-based diet quality [14], dietary guideline index [12], the Mediterranean-DASH Intervention for Neurodegenerative Delay (MIND) [12], and dietary inflammatory index [12]. Nevertheless, some prospective studies have shown a positive change in MCS with the consumption of healthy diets and this association is more pronounced among women, particularly younger women [14], compared with men [12]. Furthermore, consistent with our findings, decreased adherence to healthy dietary patterns such as the Dutch Healthy Diet index, high fiber diet, and vegetarian diet was associated with worse physical health [10, 11]. This finding was not observed in other studies [12, 13]. It should be noted that cross-sectional studies reported the most positive association between a healthy diet and improvements in both mental and physical health [8, 10, 13], however, prospective studies with short follow-up periods (less than 5-year period) showed conflicting results [14, 41] and those with over 12-year follow-ups did not observe this association [12, 42].

The current study did not find any association between UPF and MCS. In contrast to our findings, a recent meta-analysis of five observational studies revealed that HRQOL was significantly worse in individuals with high adherence to unhealthy dietary patterns or a Western dietary pattern [43]. Furthermore, the consumption of fast food, sweets, carbonated beverages, and salty snacks was linked to a lower quality of life in children and adolescents in another meta-analysis [44]. It is important to note that significant heterogeneities were observed in earlier research included in these meta-analyses. Evidence from prospective and cross-sectional studies in Europe has shown a harmful association between unhealthy dietary patterns and MCS [45–47]. The 4-year Follow-up SUN Project study in Spain demonstrated a significant inverse dose-response association between adherence to the Western dietary pattern - characterized by high consumption of fast food, red and processed meats, high-fat dairy products, processed foods, refined grains, eggs, commercial bakery goods, and sauces- and MCS score and its subscales such as vitality, social functioning, role emotional, and mental health [45]. In another population-based 12-year longitudinal study involving Australian adults aged 60 years and older, higher intakes of red meat protein (but not processed animal protein, and other animal protein), were associated with detrimental changes in MCS. Specifically, for every additional 10 g of red meat protein consumed, there was a 0.4-point deterioration in MCS scores [47]. In a cross-sectional study involving a sample of Italian participants enrolled in the Moli-sani Project, MCS and its subscales were inversely related to eggs and sweets dietary pattern (β= −0.33; *P* for trend < 0.0001), while no significant association was observed with the meat and pasta pattern (β = 0.005; *P* for trend = 0.16) [46]. However, in two randomized control trials, the consumption of a high-protein diet including 160 g/d lean red meat did not affect MCS during the intervention [48–50]. Most of the studies mentioned were conducted in Europe, where dietary habits and quality of life differ from those in the Middle East and North Africa (MENA) region. The consumption of ultra-processed foods in these studies, which observed the association between the Western dietary pattern and MCS [45–47], was notably higher compared to that in our study. In these studies, ultra-processed food consumption varied from 17% of total energy intake in the Moli-sani Study involving the Italian population [51] to 42% in the Australian Health Survey [52], which exceeded the median intake in our study (11.9%).

Socioeconomic status influences the dietary choices [53, 54]. There are reported differences in consumption of ultra-processed foods based on socio-economic status, with higher consumption among those with high socioeconomic status in both developed and developing countries [15, 55–57]. However, some studies, such as the UK National Diet and Nutrition Survey and NHANES, showed no differences in ultra-processed foods intake across different socioeconomic categories [16, 17, 58], and even, data from the 2008–2016 UK NDNS indicated lower ultra-processed foods consumption in higher social class groups [17], possibly due to the greater affordability of these foods in more developed countries [54]. Additionally, the concomitance of healthy and unhealthy eating habits was reported among the most favored social segments [55, 56]. In the current study, ultra-processed food consumption was found to be higher among lower-educated individuals and non-employed populations, which is consistent with some studies [4], but not all [59]. Socioeconomic conditions can also affect quality of life, although there are health disparities related to socioeconomic factors across different countries. In joint Canada/United States Survey of Health conducted in 2002–2003 found that income was associated with HRQoL in older adults in the United States, but not in Canada, after adjustment for confounders [18, 19]. Similarly, in Germany and Sweden, both physical and mental HRQOL are influenced by income and educational levels [12, 60], but this association was negligible in the Netherlands [20]. Patients with chronic renal failure who are unemployed, have low educational levels and have poor QOL [61]. In the current study, we found that level of education, but not occupation status, is positively associated with PCS. Occupation status modified the association between ultra-processed foods and both physical and mental HQL, and this association was found only among non-employed subjects. It could be possible that both diet and HRQoL are linked to socioeconomic status, which may impact access to food and health care [10]. Furthermore, education levels modified the association between ultra-processed foods and physical HRQoL, and this association was significant only among lower-educated individuals.

Smoking resulted in a significant decrease in PCS over time [21, 22], and a dose-response relationship was found between the amount of smoking and impaired HRQoL [62, 63]. Conversely, quitting smoking was inversely associated with physical health distress [22], and this finding can be extrapolated to different socioeconomic and cultural groups [62]. Muscle weakness caused by substances inhaled in cigarettes may decrease the quality of life [22]. However, the relationship between smoking status and low mental health is debated [21, 63]. A better mental quality of life was observed among smokers [63], however in another study, continuing smokers for 8 years led to improvements in MCS [21]. The observed association between smoking and low physical health and, in some studies, mental health may be due to poor nutritional quality. Smoking was linked to higher consumption of alcoholic beverages, coffee, sweets, and ultra-processed foods. [24–26]. As mentioned earlier, there is a positive relationship between ultra-processed food consumption and poor quality of life. However, to our knowledge, no study has examined the effect of smoking on the relationship between ultra-processed foods and quality of life. A previous study reported a combined effect of smoking and ultra-processed foods on colorectal adenomas [27], consistent with the findings of this study.

HRQoL’s benefit of physical activity is well documented [64]. Sedentary lifestyle habits were positively associated with higher consumption of ultra-processed foods [59, 65], and poor mental and physical QoL [12, 23, 66] but results have been inconclusive [13]. In the current study we found that although there is no difference in ultra-processed foods consumption between active and inactive subjects, the physical well-being declined as ultra-processed foods consumption increased among inactive subjects.

Our findings have shown that the consumption of ultra-processed foods reduces PCS in the total population, as well as among individuals with low physical activity, smokers, and those with low socioeconomic status. Unhealthy dietary fats have been identified as risk factors for muscle loss and physical disability [67, 68]. Dietary fats serve as a major energy source for muscles, and a strong association has been observed between dietary fat intake and sarcolemma (muscle membrane), highlighting the impact of dietary fat on muscle composition [67]. Ultra-processed foods such as hydrogenated vegetable oil, mayonnaise, margarine, fried foods, and baked goods are major sources of saturated fatty acids, Trans fatty acids, and cholesterol. These unhealthy dietary fats have been linked to muscle loss through the induction of insulin resistance and low-grade inflammation (circulating cytokines). Muscle mass loss is associated with functional impairments, physical disability, frailty, fractures, and a diminished quality of life [67, 68]. However, in our current study, the consumption of ultra-processed foods was not associated with PCS among individuals with healthy lifestyles and high socioeconomic status, suggesting that these factors may mitigate the adverse effects of ultra-processed foods on disability by preventing insulin resistance and inflammation. Nevertheless, additional research is necessary to confirm the findings. Moreover, an unhealthy diet can lead to obesity and other chronic diseases, such as diabetes, which can also have a detrimental impact on mental health in the long term [69]. Further investigations are warranted to elucidate the potential association between diet and both physical and mental well-being.

This study has several strengths. It is the first investigation to assess the combined effect of ultra-processed foods, lifestyle, and socioeconomic factors on HRQoL. Dietary intake was evaluated using a valid and reliable FFQ, a gold standard tool in assessing habitual dietary intake. Additionally, we used the alternative approach for assessing dietary intake, aiming to reduce within-subject variability and evaluate the long-term diet more concisely. Furthermore, by conducting this study in the Middle East and North Africa region with different dietary habits and HRQoL compared to Western and Eastern countries, we can expand our knowledge about this association. However, the generalizability of our findings to other populations needs to be done with caution due to differences in the association between ultra-processed food consumption and HRQoL across different lifestyles and socioeconomic statuses. Another limitation is the potential presence of residual or unmeasured confounders. Furthermore, as our study is observational, we are unable to establish causality. Additionally, the present findings were based on a 3-year follow-up period; prospective studies with a longer follow-up duration are needed to substantiate our conclusions. Finally, our study has limited data on other lifestyle factors (such as alcohol consumption) and socioeconomic factors (like disposable income). Further, longitudinal studies are essential to elucidate the effect of these lifestyle and socioeconomic factors on the association between dietary variables and HRQOL.

## Conclusions

Consuming unhealthy foods such as ultra-processed foods reduced physical well-being, particularly in women and among individuals with low socioeconomic status and unhealthy behaviors. This finding highlights the importance of adopting appropriate lifestyle habits along with a healthy diet among women and subjects with low socioeconomic status.

## Data Availability

The datasets generated and/or analysed during the current study are not publicly available due institution’s policy but are available from the corresponding author on reasonable request.

## Abbreviations

HRQoL: Health-related quality of life
PCS: Physical component summary
MCS: Mental component summary
GLM: General linear models
TLGS: Tehran Lipid and Glucose Study
FFQ: Food Frequency Questionnaire
RIES: Research Institute for Endocrine Sciences
FCT: Food Composition Table
MAQ: Modifiable Activity Questionnaire
MET: Metabolic-equivalent
MET-min/week: Metabolic-equivalent minutes per week
SF-12v2: Short-form 12-item health survey version 2
SE: Standard error
