# Correction: The association between ultra-processed food consumption and health-related quality of life differs across lifestyle and socioeconomic strata

**DOI:** 10.1186/s12889-024-20189-2

**Published:** 2024-10-02

**Authors:** Somayeh Hosseinpour-Niazi, Mahdieh Niknam, Parisa Amiri, Parvin Mirmiran, Elaheh Ainy, Neda Izadi, Zahra Gaeini, Fereidoun Azizi

**Affiliations:** 1 Nutrition and Endocrine Research Center, Research Institute for Endocrine Sciences, Shahid Beheshti University of Medical Sciences, P.O. Box: 19395-4763, No. 24, A’rabi St., Yeman Av., Velenjak, Tehran, Iran; 2 Research Center for Social Determinants of Health, Research Institute for Endocrine Sciences, Shahid Beheshti University of Medical Sciences, P.O. Box: 19395-4763, No. 24, A’rabi St., Yeman Av., Velenjak, Tehran, Iran; 3 Department of Clinical Nutrition and Dietetics, Faculty of Nutrition Sciences and Food Technology, National Nutrition and Food Technology Research Institute, Shahid Beheshti University of Medical Sciences, Tehran, Iran; 4 Safety Promotion and Injury Prevention Research Center, Shahid Beheshti University of Medical Sciences, Tehran, Iran; 5 Endocrine Research Center, Research Institute for Endocrine Sciences, Shahid Beheshti University of Medical Sciences, Tehran, Iran

**BMC Public Health (2024) 24:1955**

10.1186/s12889-024-19351-7

In the original publication of this article there was an incorrect author name. The incorrect and correct information is listed in this correction article, the original article has been updated.

Incorrect

Elaheh Einy

Correct

Elaheh Ainy

